# Urinary Neutrophil Mediators as Predictive Biomarkers for Severe Dengue in Adults

**DOI:** 10.1093/ofid/ofaf813

**Published:** 2026-01-09

**Authors:** Andrew Teo, Po Ying Chia, Sharlene Ho, Tsin Wen Yeo

**Affiliations:** Department of Respiratory and Infectious Diseaes, Lee Kong Chian School of Medicine,Nanyang Technological University, Singapore, Singapore; Department of Medicine, The Doherty Institute, University of Melbourne, Melbourne, Australia; National Centre for Infectious Diseases, Singapore, Singapore; Department of Respiratory and Infectious Diseaes, Lee Kong Chian School of Medicine,Nanyang Technological University, Singapore, Singapore; National Centre for Infectious Diseases, Singapore, Singapore; Department of Infectious Diseases, Tan Tock Seng Hospital, Singapore, Singapore; Department of Respiratory and Critical Care Medicine, Tan Tock Seng Hospital, Singapore, Singapore; Department of Respiratory and Infectious Diseaes, Lee Kong Chian School of Medicine,Nanyang Technological University, Singapore, Singapore; Woodlands Health, National Healtcare Group, Singapore, Singapore

**Keywords:** arbovirus, neutrophil gelatinase-associated lipocalin, neutrophils, severe dengue, soluble urokinase plasminogen activator receptor

## Abstract

Reliable prognostic biomarkers remain an unmet need in dengue. We evaluated urinary neutrophil gelatinase-associated lipocalin and soluble urokinase plasminogen activator receptor for severe dengue prediction in the febrile phase. Urinary neutrophil gelatinase-associated lipocalin and soluble urokinase plasminogen activator receptor demonstrated areas under the receiver operating curve of 0.88 and 0.79, respectively, in discriminating against severe dengue. The noninvasive sampling offers practical advantages as risk stratification tools.

Dengue is the most prevalent global arboviral infection, with over half the world's population at risk [1]. The number is projected to rise significantly due to increased urbanization and geographic spread of the mosquito vector *Aedes aegypti*. In 2023, the World Health Organization (WHO) recorded the highest number of dengue cases at 6.5 million, but this figure was surpassed in 2024, with >14 million cases documented [2].

Most patients with dengue experience a febrile phase followed by full recovery. However, 2% to 5% may develop severe dengue (SD) marked by vascular leakage, bleeding, or organ dysfunction with a fatality rate of 5% [1]. Optimal dengue management requires accurate prediction of SD, and current WHO guidelines use “warning signs” to recommend hospitalization. Although warning signs have been validated as predictors of SD, their high sensitivity but poor specificity lead to inconsistent interpretation and potentially unnecessary hospitalization during large outbreaks, which can overwhelm health care facilities [3]. Prognostic biomarkers that are deployable across a variety of health care settings could mitigate this strain by improving risk stratification and resource allocation during outbreaks [4, 5].

Dysregulated neutrophil responses contribute to SD, and we previously linked blood neutrophil mediators, including myeloperoxidase and soluble urokinase plasminogen activator receptor (suPAR), with SD in adults [4, 6, 7]. Neutrophil gelatinase-associated lipocalin (NGAL), a protein secreted by activated neutrophils, was associated with infection in sera but not disease severity [8]. However, measuring these biomarkers in blood is logistically challenging in low- and middle-income countries where dengue is endemic. Urinary biomarkers could offer a convenient alternative, and urinary levels of NGAL and suPAR have demonstrated utility as biomarkers of acute kidney illness in critical illness [9, 10]. Yet, their utility in urine as predictive biomarkers of SD remain unexplored. Here, we assessed the utility of urinary NGAL (uNGAL) and urinary suPAR (usuPAR) as predictive biomarkers of SD in the febrile phase of adult patients with dengue.

## METHODS

### Participants

Patient recruitment details were previously reported [4, 6]. Briefly, this prospective observational study enrolled patients aged >16 years at 3 time points: the febrile, critical, and recovery phases, with plasma and urine samples collected at median days 4, 6, and 22, respectively. Patients were categorized by WHO 2009 classifications: dengue without warning signs (DwoWS), dengue with warning signs (DWS), and SD [11]. In addition to disease severity, demographics and laboratory information (hematocrit, platelet counts, liver enzymes, and albumin) were collected. The critical phase was defined according to the day with the lowest platelet count concurrent with the highest hematocrit and defervescence. The maximal change in hematocrit, a measure of vascular leakage, was calculated by using the following formula: (highest hematocrit level in critical – hematocrit level taken prior to discharge) / hematocrit level on discharge × 100. Controls were adults with no febrile episodes 2 weeks prior to recruitment and no previous dengue within the past 6 months.

### Urinary Protein Measurements

Urinary concentrations of NGAL (#DY1757, Lot: P432322) and suPAR (#DY807, Lot: P433906; R&D System DuoSet) were assayed by enzyme-linked immunosorbent assays based on the manufacturer's protocols. We assayed 319 urine samples: 113 at the febrile phase, 102 at the critical phase, 74 at the recovery phase, and 30 healthy controls. Among these, 97 were paired samples from the febrile and critical phases. Additionally, 39 samples from febrile-phase plasma were assayed for NGAL.

### Statistical Methods

Analysis of variance or the Kruskal-Wallis test was performed to determine intergroup differences for parametric or nonparametric continuous variables, respectively. Categorical variables were assessed by the χ^2^ test. Post hoc pairwise comparisons were used to compare differences among DwoWS, DWS, and SD. Spearman methods were used to determine correlation coefficients between laboratory measures of severity and uNGAL/suPAR. The area under the receiver operating curve (AUROC) and corresponding 95% CI were calculated to evaluate the suitability of uNGAL and usuPAR as potential biomarkers for SD in the febrile phase. For mediators with an AUROC ≥0.7, a Youden index was calculated to determine an optimal cutoff for SD. The negative predictive value (NPV), positive predictive value (PPV), sensitivity, and specificity were also determined. Additionally, PPVs were calculated by determined cutoff values with the expected prevalence of SD in patients admitted for inpatient management in our institution and affiliated primary care settings.

### Ethical Review

This study was approved by the National Healthcare Group Domain Specific Review Board (E/2016/00982). Written informed consent was obtained from all patients prior to enrollment.

## RESULTS

Detailed baseline and clinical characteristics are presented in Table 1. There were 125 patients recruited for the study: 40 with DwoWS, 44 with DWS, 11 with SD, and 30 controls. For the DwoWS, DWS, and SD groups, 32, 34, and 10 patients were enrolled in the febrile phase, respectively.

**Table 1.** Patients’ Baseline Characteristics

	No. (%) or Median (IQR) [Range]				
	Controls (n = 30)	DwoWS (n = 40)	DWS (n = 44)	SD (n = 11)	*P* Value^a^
Male	15 (50.0)	16 (64.0)	15 (62.5)	6 (60)	.252
Age, y	44 (32–59) [23–75]	40 (32–55) [24–73]	48 (33–61) [22–80]	61 (36–68) [24–83]	.07
BMI, kg/m^2^	24.3 (21.9–26.4)	27.2 (22.9–30.7)	25.9 (23.3–30.7)	26.2 (23.5–28.7)	.392
CCI	0 (0–0) [0–2]	0 (0–0) [0–3]	1 (0–0) [0–3]	1.5 (0–1) [0–5]	.072
Diabetes mellitus	3 (10)	4 (9.1)	8 (15.7)	3 (25)	.423
Hypertension	4 (13.3)	9 (20.5)	14 (27.5)	9 (75)	.**001**
Previous dengue	3 (10)	1 (2.3)	2 (3.9)	2 (16.7)	.132
Day of illness					
For febrile phase	…	4 (3–5)	4 (3–5)	4 (4–5)	.868
For critical phase	…	6 (5–7)	6 (5–7)	6 (5.5–7)	.277

Abbreviations: BMI, body mass index; CCI, Charlson Comorbidity Index; DwoWS, dengue without warning signs; DWS, dengue with warning signs; NGAL, neutrophil gelatinase-associated lipocalin; SD, severe dengue.

^a^Analysis of variance/Kruskal-Wallis or χ^2^/Fisher exact for comparison of controls, DwoWS, DWS, and severe dengue groups. Bold indicates *P* < .05.

### uNGAL and usuPAR Are Increased in Dengue Cases vs Controls

uNGAL and usuPAR levels were significantly higher in dengue cases as compared with controls and in SD cases as compared with nonsevere cases in the febrile and critical phases (Table 2). Plasma NGAL was measured in a subset of controls and patients with dengue with no differences found between the groups.

**Table 2.** Longitudinal Urinary NGAL and suPAR Levels

	Levels, pg/mL, Median (IQR); No.				
	Controls (n = 30)	DwoWS (n = 40)	DWS (n = 44)	SD (n = 11)	*P* Value^a^
Urinary NGAL					
Febrile phase	633 (267–1990)	2327 (1533–3870)	1734 (584–3692)	5246 (5000–7242)	.**010**
Critical phase	…	2130 (1108–5178)	2800 (1777–5097)	3576 (2508–8704)	.**001**
Recovery phase	…	1666 (143–3615); 6	1620 (617–2135); 10	1320 (1136–2178); 6	.9
Urinary suPAR					
Febrile phase	2023 (738–2495)	6387 (3711–10 661)	5251 (2535–8294)	13 877 (7597–15 514)	.**003**
Critical phase	…	13 779 (5192–19 215)	9752 (4469–17 041)	23 778 (9567–26 561)	.**001**
Recovery phase	…	1604 (1366–2204); 6	1624 (259–3605); 10	10 789 (6149–19 806); 6	.06
Plasma NGAL					
Enrollment	33 530 (26 497–42 909); 8	29 856 (14 985–52 759); 10	24 874 (18 297–33 888); 10	31 190 (20 081–48 129); 11	.80
Hypertensive cases					
Urinary NGAL					
Febrile phase	…	3185 (2117–4500); 6	2322 (1407–4128); 8	5820 (5000–7769); 8	**.02**
Critical phase	…	4139 (2208–5739); 9	3770 (1496–9313); 14	3676 (2748–9204); 9	.3
Urinary suPAR					
Febrile phase	…	7603 (5634–11 851); 6	5912 (2961–8457); 8	14 190 (11 374–15 887); 8	**.02**
Critical phase	…	14 892 (13 543–15 700); 9	13 838 (8141–16 777); 14	25 350 (19 285–26 976); 9	**<.001**

Abbreviations: DwoWS, dengue without warning signs; DWS, dengue with warning signs; NGAL, neutrophil gelatinase-associated lipocalin; SD, severe dengue; suPAR; soluble urokinase plasminogen activator receptor.

^a^Analysis of variance/Kruskal-Wallis for comparison of controls, dengue without warning signs, dengue with warning signs, and severe dengue groups. Bold indicates *P* < .05.

### uNGAL as a Prognostic Biomarker of SD in the Febrile Phase

In the febrile phase, the AUROC of uNGAL to predict SD was 0.88 (95% CI, .79–.96). The Youden index for uNGAL was 3530 pg/mL with a sensitivity of 0.99 and specificity of 0.74. In the inpatient setting with an SD prevalence of 16.5%, the PPV was 43% and the NPV was 99%. In the outpatient setting with an SD prevalence of 8.8%, the PPV was 27% and the NPV was 99%.

### usuPAR as a Prognostic Biomarker of SD in the Febrile Phase

In the febrile phase, the AUROC of usuPAR to predict SD was 0.79 (95% CI, .65–.95). The Youden index for usuPAR was 13 453 pg/mL with a sensitivity of 0.65 and specificity of 0.93. In the inpatient setting with an SD prevalence of 16.5%, the PPV was 31% and the NPV was 90%. In the outpatient setting with an SD prevalence of 8.8%, the PPV was 20% and the NPV was 95%.

### uNGAL and suPAR and Laboratory Biomarkers of Severity

In the critical phase, there were significant associations between uNGAL and maximal hematocrit change (*r* = 0.36, *P* = .003), aspartate aminotransferase (*r* = 0.30, *P* = .03), alanine aminotransferase (*r* = 0.26, *P* = .04), and albumin (*r* = −0.29, *P* = .005). There were also significant associations with maximal hematocrit change and usuPAR in the febrile (*r* = 0.28, *P* = .01) and critical (*r* = 0.23, *P* = .02) phases but not with the other biomarkers. There was no association between platelet counts and uNGAL/usuPAR in either the febrile or critical phase.

## DISCUSSION

In a prospective cohort of Asian adults with dengue, urinary biomarkers NGAL and suPAR were significantly elevated in dengue cases as compared with healthy controls, with concentrations rising progressively with disease severity. Importantly, in an inpatient setting during the febrile phase, uNGAL demonstrated a PPV of 43% and a NPV of 99%, while usuPAR had a PPV of 31% and NPV of 90% in discriminating against severe disease. In an outpatient setting where the prevalence of SD was lower, the febrile-phase uNGAL had a PPV of 27% and NPV of 99%, while usuPAR had a PPV of 20% and NPV of 95%.

Effective dengue management requires biomarkers that predict SD early [3, 4]. Current WHO 2009 dengue warning signs, used for hospital admission, have high sensitivity (96%) but poor specificity (18%) for predicting SD [11]. Importantly, the low PPV (15%) may result in unnecessary admissions and resource strain [3, 12]. Multiple studies have been done to evaluate viral, inflammatory, and endothelial markers as potential prognostic biomarkers of SD [13]. However, most of these assays require blood sampling and specialized laboratory equipment, which can be costly in resource-constrained countries. Urinary biomarkers offer an attractive alternative, eliminating the need for venipuncture and associated equipment. To our knowledge, only one previous study has evaluated the use of a urinary biomarker to predict SD, by measuring leukotriene 4 in the urine of patients with dengue. It was elevated in severe cases but had only a modest predictive value (AUROC, 0.67) of severe complications [14]. Our findings indicate that uNGAL has a superior PPV for SD in an inpatient setting as compared with warning signs alone. While usuPAR demonstrates a lower PPV than uNGAL, it still outperforms warning signs alone in predictive capacity. In contrast, the predictive performance of these urinary biomarkers was lower than that of our previous reported blood-based biomarkers, suPAR and soluble suppressor of tumorogenicity 2, possibly reflecting biological differences between blood and renal proteins [4, 5]. Together, our results suggest that measurement of uNGAL and, albeit to a lesser extent, usuPAR when combined with warning signs could significantly reduce unnecessary hospital admissions without an increased risk of misdiagnosis of severe cases.

This study is limited by its small cohort of older hospitalized adults with SD. Future validation in primary care and pediatrics populations is needed.
